# Associations between Life’s Essential 8 and abdominal aortic calcification among US Adults: a cross-sectional study

**DOI:** 10.1186/s12889-024-18622-7

**Published:** 2024-04-19

**Authors:** Quanjun Liu, Hong Xiang, Shuhua Chen, Jie Ouyang, Huiqin Liu, Jing Zhang, Yanfei Chai, Peng Gao, Xiao Zhang, Jianing Fan, Xinru Zheng, Hongwei Lu

**Affiliations:** 1 Health Management Center, The Third Xiangya Hospital of Central South University, Changsha, China; 2 Department of Cardiology, The Third Xiangya Hospital of Central South University, No. 138, Tongzipo Road, Yuelu District, Changsha, China; 3 Center for Experimental Medicine, The Third Xiangya Hospital of Central South University, Changsha, China; 4 Department of Biochemistry, School of Life Sciences of Central, South University, Changsha, China

**Keywords:** Cardiovascular health, Life’s Essential 8, Abdominal aortic calcification, Cardiovascular disease, NHANES

## Abstract

**Background:**

Cardiovascular health (CVH) and abdominal aortic calcification (AAC) are closely linked to cardiovascular disease (CVD) and related mortality. However, the relationship between CVH metrics via Life’s Essential 8 (LE8) and AAC remains unexplored.

**Methods:**

The study analyzed data from the 2013–2014 National Health and Nutrition Examination Survey (NHANES) cohort, which included adults aged 40 or above. The research used the LE8 algorithm to evaluate CVH. Semi-quantitative AAC-24 scoring techniques were employed to assess AAC, categorized into no calcification, mild to moderate calcification, and severe calcification.

**Results:**

The primary analysis involved 2,478 participants. Following adjustments for multiple factors, the LE8 score exhibited a significant association with ACC risk (Mild-moderate ACC: 0.87, 95% CI: 0.81,0.93; Severe ACC: 0.77, 95% CI: 0.69,0.87, all *P* < 0.001), indicating an almost linear dose–response relationship. Compared to the low CVH group, the moderate CVH group showed lower odds ratios (OR) for mild-moderate and severe calcification (OR = 0.78, 95% CI: 0.61–0.99, *P* = 0.041; OR = 0.68, 95% CI: 0.46–0.99, *P* = 0.047, respectively). Moreover, the high CVH group demonstrated even lower ORs for mild-moderate and severe calcification (OR = 0.46, 95% CI: 0.31, 0.69, *P* < 0.001; OR = 0.29, 95% CI: 0.14, 0.59, *P* = 0.001, respectively). Interactions were found between chronic kidney disease (CKD) condition, history of CVD, marital status and CVH metrics to ACC. Participants without CKD exhibited a more pronounced negative association between the CVH metric and both mild-moderate and severe ACC. Those lacking a history of CVD, and never married/widowed/divorced/separated showed a stronger negative association between the CVH metric and severe ACC.

**Conclusions:**

The novel CVH metrics demonstrated an inverse correlation with the risk of AAC. These findings suggest that embracing improved CVH levels may assist in alleviating the burden of ACC.

**Supplementary Information:**

The online version contains supplementary material available at 10.1186/s12889-024-18622-7.

## Introduction

Abdominal aortic calcification (AAC) refers to the accumulation of calcium deposits in the abdominal aorta, the largest artery in the abdominal cavity [1]. This buildup within the arterial wall can render the aorta rigid and potentially narrow its passageway. Furthermore, these deposits contribute to the formation of atherosclerotic plaques, where lipid plaques accumulate and calcify along blood vessel walls, ultimately triggering cardiovascular disease (CVD) [2, 3]. Numerous prospective studies have affirmed the link between the severity of AAC and the incidence of CVD, including coronary artery disease, myocardial infarction, stroke, and increased mortality rates [4–6]. Managing and preventing AAC is critical and desirable in reducing the prevalence and occurrence of cardiovascular disease.

In 2010, the American Heart Association (AHA) introduced Life’s Simple 7 (LS7), a set of cardiovascular health (CVH) metrics encompassing seven key behavioral and health factors: healthy diet, physical activity, normal body mass index (BMI), no smoking, normal blood pressure, normal fasting glucose, and normal total cholesterol [7]. Subsequent extensive research has shed light on the strengths and limitations of this initial approach in defining and quantifying cardiovascular health. Consequently, the AHA has recently updated its CVH assessment tool to Life’s Essential 8 (LE8), a scoring system more attuned to individual variations, emphasizing the significance of social factors and mental health in determining CVH [8]. Higher CVH scores, as assessed by LS7/LE8, have been correlated with a reduced risk of CVD and related mortality [9–11].

Given the established correlation between LE8 and AAC with cardiovascular disease and cardiovascular death, we hypothesize that ideal CVH is associated with lower severity of AAC. Several lifestyle and clinical measures, such as a heart-healthy diet, physical activity, smoking status, sleep pattern, blood glucose, blood lipid, and blood pressure, have been associated with AAC risk [12–16]. However, there remains unexplored territory concerning the relationship between CVH metrics evaluated through the LE8 score and AAC.

To explore the association of novel CVH metrics, and its components, with AAC, we first conducted an observational study utilizing data from the National Health and Nutrition Examination Survey (NHANES) cohort of 2013–2014.

## Methods

### Study design and participants

**Fig. 1:**
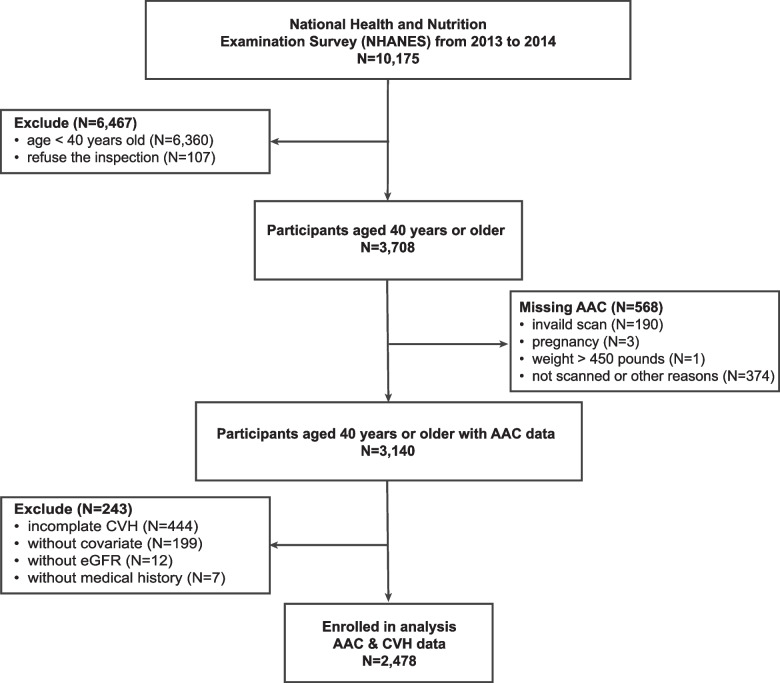
Screening flow of participants included in the research. Abbreviation: ACC, abdominal aortic calcification; CVH, cardiovascular health; eGFR, estimated glomerular filtration rate

This population-based, cross-sectional study was performed based on the NHANES project conducted by the National Center for Health Statistics at the Centers for Disease Control and Prevention (CDC) in the United States. The survey protocol received approval from the NCHS institutional review board, and all respondents provided written informed consent. All NHANES data utilized in this analysis are publicly available at https://www.cdc.gov/nchs/nhanes. As the dual-energy X-ray absorptiometry (DXA) scan was only conducted in 2013–2014 in NHANES, participants from this period were included [17]. After excluding participants below 40 years old (*n* = 6,360), refusal for inspection (*n* = 107), pregnancy (*n* = 3), overweight (weight > 450 pounds) individual (*n* = 1), invalid scans (*n* = 190), and those not scanned for other reasons (*n* = 374), 3140 participants with complete AAC data were included. Further exclusion criteria involved participants with missing CVH metrics data (*n* = 444), missing key covariates (*n* = 199), missing estimated glomerular filtration rate (eGFR) data (*n* = 12), and those without relevant medical history (*n* = 7). Ultimately, 2,478 adult participants were included in the primary analysis (Fig. 1).

### Measurement of LE8

The components of LE8 encompass diet, physical activity, nicotine exposure, sleep health, body mass index (BMI), blood lipids, blood glucose, and blood pressure (BP), categorized into health behaviors (diet, physical activity, nicotine exposure, sleep) and health factors (BMI, blood lipids, blood glucose, BP) [8]. Each metric involves a scoring algorithm ranging from 0 to 100 points. Detailed algorithms for calculating the LE8 scores based on NHANES data have been previously published and can be accessed in Table S1. According to AHA recommendations, overall CVH scores falling within 80 to 100 are considered high CVH, 50 to 79 signify moderate CVH, and 0 to 49 points reflect low CVH.

The Healthy Eating Index (HEI) 2015 was used to assess the diet metrics [18]. Participants’ dietary intakes collected from two 24-h dietary recalls were combined with the United States Department of Agriculture (USDA) food patterns equivalents data to establish and compute the HEI-2015 scores. The HEI-2015 comprised 12 components, measuring dietary adequacy (intakes of entire fruit, whole fruit, total vegetables, greens and beans, whole grains, dairy, complete protein foods, seafood, and plant proteins, and fatty acids), where higher scores indicated increased consumption. Additionally, three components gauged moderate consumption of refined grains, sodium, and empty calories (solid fats, alcohol, and added sugars), with higher scores indicating lower consumption. Physical activity, smoking habits, sleep time, diabetes, and medication history were obtained via self-report questionnaires. BP, weight, and height measurements were acquired in the mobile examination center using standard methods [19]. BMI was calculated as weight/height^2^ from these measurements. Laboratory tests were conducted to assess blood lipids, blood glucose, and Hemoglobin A1c.

### Abdominal aortic calcification

Each participant’s scan and phantom scan underwent analysis by the UCSF using standard radiologic techniques and study-specific NHANES protocols. AAC-24 scoring semi-quantitative techniques were employed for ACC assessment [20]. The scoring method involved dividing the anterior and posterior aortic walls into four segments, corresponding to the areas in front of the lumbar vertebrae L1-L4. Scores were obtained separately for these walls, resulting in a range from “0” to “6” for each vertebral level and “0” to “24” for the total score. Higher AAC scores indicated a more severe calcification condition in the abdominal aorta. AAC scores were categorized into three groups: no calcification (AAC = 0), mild to moderate calcification (0 < AAC ≤ 6), and severe calcification (AAC > 6) [21, 22].

### Demographic characteristics and other covariate

Race/ethnicity (non-Hispanic (NH) white, NH black, Hispanic, other race) were categorized based on the survey design. To simplify the interpretation of results, education level was simplified into below high school (less than 11th grade), high school graduate or general educational development test (GED) (high school Grad/GED), and some college or above (AA degree or College or above). And marital status was divided into married or living with a partner, and never married/widowed/divorced/separated. The Poverty-Income Ratio (PIR) served as an index of income related to federally established poverty thresholds, accounting for economic inflation and family size. eGFR was calculated using the Chronic Kidney Disease-Epidemiology Collaboration (CKD-EPI) equation [23]. Chronic kidney disease (CKD) was defined as an eGFR of 15–59 mL/min/1.73 m^2^, corresponding to stages 3–4. A history of CVD included self-reported coronary heart disease, heart attack, and stroke.

### Statistical analysis

Categorical variables were presented as frequency (percentages) and compared using chi-square tests. Continuous variables followed a normal distribution and were presented as mean ± standard deviation (SD), analyzed via the Kruskal–Wallis H test.

Multivariable logistic regressions were employed to independently assess the association of novel CVH metrics with AAC, adjusting for potential demographic confounders (age (as a continuous variable), gender and race/ethnicity, poverty ratio (as a continuous variable), education levels, and marital status) using forward selection methods. Odds ratios (ORs) were calculated in an unadjusted model, age, gender and race/ethnicity-adjusted model (Model 1), and after adjustment for potential confounders, including age, gender, race/ethnicity, poverty ratio, education levels, and marital status (Model 2). Additionally, multifactorial logistic regression analysis was conducted to explore correlations between the components of CVH metrics and ACC, adjusting for the aforementioned factors and other constituents of CVH metrics.

The restricted cubic spline regression examined the potential nonlinear relationships between the LE8 score and ACC, with nonlinearity tested using the likelihood ratio test. Subsequently, stratification and interaction analyses were performed by gender, age, race, marital status, CKD condition, and history of CVD. All statistical analyses were conducted using IBM SPSS Statistics 27 and R software. A two-sided *p*-value < 0.05 was considered statistically significant.

## Results

### Baseline characteristics of the study population

The characteristics and CVH of participants across the three categories of total ACC score (no, mild-moderate, severe) are shown in Table 1. Notably, 70.4% of participants had no ACC, 20.6% had mild-moderate ACC, and 9.0% had severe ACC. Those with higher ACC scores were notably older, more likely to be NH white, and never married/widowed/divorced/separated (*P* < 0.05). Furthermore, individuals with a history of CVD, diabetes, stroke, and CKD were more likely to have higher ACC scores (all *P* values < 0.05). Concerning CVH, higher ACC score participants displayed lower LE8, physical activity, nicotine exposure, blood glucose, and blood pressure scores but higher BMI scores (all *P* values < 0.05). However, diet, sleep health, and blood lipid scores did not significantly differ among the three groups (*P* > 0.05).

**Table 1:** Characteristics and cardiovascular health assessment based on no, mild-moderate and severe abdominal aortic calcification

Characteristics	Total *N* = 2478	No ACC *N* = 1744	Mild-moderate ACC *N* = 511	Severe ACC *N* = 223	*P* value
**Age, years, mean (SD)**	58.41 (11.86)	55.86 (10.89)	61.59 (11.79)	71.08 (9.19)	< 0.001
**Age groups, years, no (%)**					< 0.001
40–49	708 (28.6)	597 (34.2)	104 (20.4)	7 (3.1)	
50–59	630 (25.4)	493 (28.3)	116 (22.7)	21 (9.4)	
60–69	618 (24.9)	428 (24.5)	140 (27.4)	50 (22.4)	
≥ 70	522 (21.1)	226 (13.0)	151 (29.5)	145 (65.0)	
**Male, no (%)**	1196 (48.3)	833 (47.8)	258 (50.5)	105 (47.1)	0.519
**Race/ethnicity, no (%)**					< 0.001
Non-Hispanic White	1153 (46.5)	745 (42.7)	261 (51.1)	147 (65.9)	
Non-Hispanic Black	481 (19.4)	366 (21.0)	88 (17.2)	27 (12.1)	
Hispanic	528 (21.3)	400 (22.9)	99 (19.4)	29 (13.0)	
Multiracial/other^a^	316 (12.8)	233 (13.4)	63 (12.3)	20 (9.0)	
**Education level, no (%)**					0.050
Below high school	518 (20.9)	355 (20.4)	104 (20.4)	59 (26.5)	
High school graduate or GED	556 (22.4)	374 (21.4)	128 (25.0)	54 (24.2)	
Some college or above	1404 (56.7)	1015 (58.2)	279 (54.6)	110 (49.3)	
**Poverty ratio, no (%)**					0.240
< 1.3	729 (29.4)	507 (29.1)	154 (30.1)	68 (30.5)	
1.3–3.5	857 (34.6)	585 (33.5)	187 (36.6)	85 (38.1)	
> 3.5	892 (36.0)	652 (37.4)	170 (33.3)	70 (31.4)	
**Marital status, no (%)**					< 0.001
Married or living with partner	1576 (63.6)	1145 (65.7)	317 (62.0)	114 (51.1)	
Never married/Widowed/divorced/separated	902 (36.4)	599 (34.3)	194 (37.9)	109 (48.9)	
**HEI-2015 diet score, mean (SD)**	55.83 (13.63)	56.03 (13.72)	55.36 (13.77)	55.39 (12.59)	0.669
**PA, min/week, mean (SD)**	698.29 (1263.22)	760.22 (1320.14)	622.92 (1213.80)	385.50 (769.53)	< 0.001
**Sleep time, h/d, mean (SD)**	6.89 (2.32)	6.85 (2.60)	6.89 (1.45)	7.25 (1.35)	< 0.001
**Body mass index, kg/m**^**2**^**, mean (SD)**	28.56 (5.66)	28.92 (5.94)	27.83 (5.03)	27.42 (4.29)	< 0.001
**Non-HDL cholesterol, mg/dL, mean (SD)**	141.28 (44.95)	142.27 (45.47)	142.93 (44.47)	129.73 (40.32)	< 0.001
**HbA1c, %, mean (SD)**	5.91 (1.16)	5.86 (1.18)	5.93 (0.99)	6.23 (1.30)	< 0.001
**SBP, mm Hg, mean (SD)**	126.95 (18.51)	125.09 (17.50)	129.78 (19.85)	134.99 (20.13)	< 0.001
**DBP, mm Hg, mean (SD)**	70.68 (12.43)	71.84 (11.83)	69.55 (13.19)	64.20 (12.99)	< 0.001
**eGFR, mL/min/1.73 m**^**2**^**, mean (SD)**	84.48 (20.26)	87.42 (18.92)	81.43 (20.22)	68.45 (21.91)	< 0.001
**CKD, no (%), mean (SD)**	272 (11.0)	119 (6.8)	76 (14.9)	77 (34.5)	< 0.001
**History of heart disease, no (%)**	129 (5.2)	53 (3.0)	32 (6.3)	44 (19.7)	< 0.001
**History of diabetes, no (%)**	403 (16.3)	250 (14.3)	85 (16.6)	68 (30.5)	< 0.001
**History of stroke, no (%)**	107 (4.3)	53 (3.0)	28 (5.5)	26 (11.7)	< 0.001
**LE8 scores (out of 100 possible points), mean (SD)**					
LE8 score	60.96 (15.02)	62.02 (15.39)	58.79 (14.22)	57.65 (12.72)	< 0.001
Diet score	39.74 (31.31)	40.07 (31.52)	38.94 (31.19)	38.97 (30.00)	0.768
Physical activity score	44.00 (46.59)	46.59 (47.03)	37.36 (44.89)	38.88 (45.26)	< 0.001
Nicotine exposure score	72.12 (37.85)	73.70 (37.51)	68.28 (39.01)	68.59 (37.17)	< 0.001
Sleep health score	80.68 (25.51)	80.17 (25.59)	80.59 (26.27)	84.93 (22.68)	0.084
Body mass index score	61.39 (31.89)	59.56 (32.82)	64.77 (29.91	68.00 (27.27)	< 0.001
Blood lipid score	61.68 (29.60)	62.22 (30.04)	59.45 (29.31)	62.51 (26.53)	0.159
Blood glucose score	70.21 (27.88)	72.19 (27.76)	67.96 (26.85)	59.82 (28.52)	< 0.001
Blood pressure score	57.84 (34.16)	61.62 (33.39)	52.94 (34.71)	39.48 (31.45)	< 0.001
**Cardiovascular health, no (%)**					< 0.001
Low (0–49)	560 (22.6)	372 (21.3)	134 (26.2)	54 (24.2)	
Moderate (50–79)	1608 (64.9)	1115 (63.9)	336 (65.8)	157 (70.4)	
High (80–100)	310 (12.5)	257 (14.7)	41 (8.0)	12 (5.4)	

Data presented as mean (standard deviation, SD) for continuous and no. (%) values for categorical

*Abbreviations: ACC* Abdominal aortic calcification, *GED* General educational development test, *HEI* Healthy eating index, *PA* Physical activity, *BMI* Body mass index, *HDL* High-density lipoprotein, *HbA1c* Hemoglobin A1c, *SBP* Systolic blood pressure, *DBP* Diastolic blood pressure, *CKD* Chronic kidney disease, *eGFR* Estimated glomerular filtration rate, *LE8* Life’s essential 8

^a^Includes non-Hispanic Asian, multi-racial, and others

### Relationship between CVH metrics and ACC

The prevalence of mild-moderate ACC was 13.2%, while severe ACC was 3.9% among high CVH participants. This was significantly lower than moderate CVH participants, where mild-moderate ACC was 20.9%, severe ACC was 9.8%, and low CVH participants, where mild-moderate ACC was 26.2% and severe ACC was 24.2%.

**Fig. 2:**
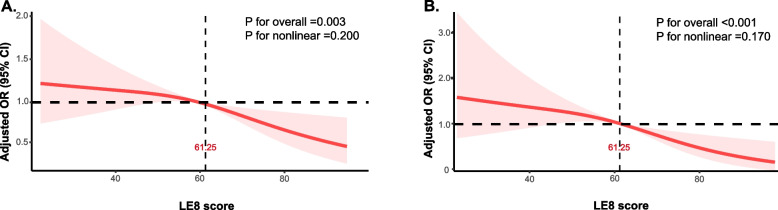
Dose–response relationships between Life’s Essential 8 score and mild-moderate (**A**) and severe (**B**) abdominal aortic calcification. Note: Odds ratio adjusted for age (as a continuous variable), gender, race, poverty ratio (as a continuous variable), education levels, and marital status. Abbreviation: OR, odds ratio; LE8, life’s essential 8

Table 2 shows results from univariate and multivariate logistic regression analyses. Unadjusted univariate logistic regression model showed an association between every 10-point increase in LE8 score and reduced OR of mild-moderate ACC (OR = 0.87, 95% confidence interval (CI): 0.81, 0.93) and severe ACC (OR = 0.83, 95% CI: 0.75, 0.91). After adjustment for age, gender, and race/ethnicity, the association between LE8 score and severity of ACC persisted across model 1. In the fully adjusted model, the impact of LE8 score was still significant when adjusting for poverty ratio, education levels, and marital status (Mild-moderate ACC: 0.87, 95% CI: 0.81,0.93; Severe ACC: 0.77, 95% CI: 0.69,0.87, both *P* < 0.001). Moreover, moderate and high CVH groups exhibited lower ORs for mild-moderate and severe calcification compared to the low CVH group after adjustments for multiple factors (*P* < 0.05). Restricted cubic spline regression analysis did not reveal nonlinear relationships between LE8 score and mild-moderate or severe ACC risk after adjustment for multiple covariates (Fig. 2) (*P* for nonlinear = 0.200, *P* for nonlinear = 0.170, respectively). Moreover, the minimal threshold for the beneficial association was 61.25 points for mild-moderate ACC and severe ACC (estimated OR = 1). Beyond this LE8 total score threshold, the risk decreases rapidly.

**Table 2:** Association of the Life’s Essential 8 scores with abdominal aortic calcification

Variables	Mild–moderate AAC versus no AAC		Severe AAC versus no AAC	
	**OR (95% CI)**	***P* value**	**OR (95% CI)**	***P* value**
**Unadjusted**				
Per 10 points increase	0.87 (0.81,0.93)	< 0.001	0.83 (0.75,0.91)	< 0.001
LE8 score				
Low (0–49)	1 (Reference)	-	1 (Reference)	-
Moderate (50–79)	0.84 (0.66,1.06)	0.132	0.97 (0.70,1.35)	0.857
High (80–100)	0.44 (0.30,0.65)	< 0.001	0.32 (0.18,0.59)	0.001
**Model 1**^**a**^				
Per 10 points increase	0.87 (0.81,0.93)	< 0.001	0.77 (0.68,0.86)	< 0.001
LE8 score				
Low (0–49)	1 (Reference)	-	1 (Reference)	-
Moderate (50–79)	0.78 (0.61,0.99)	0.041	0.68 (0.46,0.99)	0.045
High (80–100)	0.46 (0.31,0.69)	< 0.001	0.28 (0.14,0.58)	0.001
**Model 2**^**b**^				
Per 10 points increase	0.87 (0.81,0.93)	< 0.001	0.77 (0.69,0.87)	< 0.001
LE8 score				
Low (0–49)	1 (Reference)	-	1 (Reference)	-
Moderate (50–79)	0.78 (0.61,0.99)	0.041	0.68 (0.46,0.99)	0.047
High (80–100)	0.46 (0.31,0.69)	< 0.001	0.29 (0.14,0.59)	0.001

*Abbreviations*: *ACC* Abdominal aortic calcification, *OR* Odds ratio, *CI* Confidence interval, *LE8* Life’s essential 8

^a^Adjusted for age (as a continuous variable), gender and race/ethnicity

^b^Additionally adjusted for poverty ratio (as a continuous variable), education levels, and marital status

Regarding the components of LE8, physical activity, nicotine exposure, and blood lipid scores negatively correlated with mild-moderate ACC in adjusted models (all *P* values < 0.05). Similarly, nicotine exposure, blood glucose, and blood lipid scores displayed negative correlations with severe ACC (all *P* values < 0.001). In contrast, the BMI score showed a significant positive correlation with both mild-moderate and severe ACC (Table S2).

### Subgroup analysis and interaction test

**Fig. 3:**
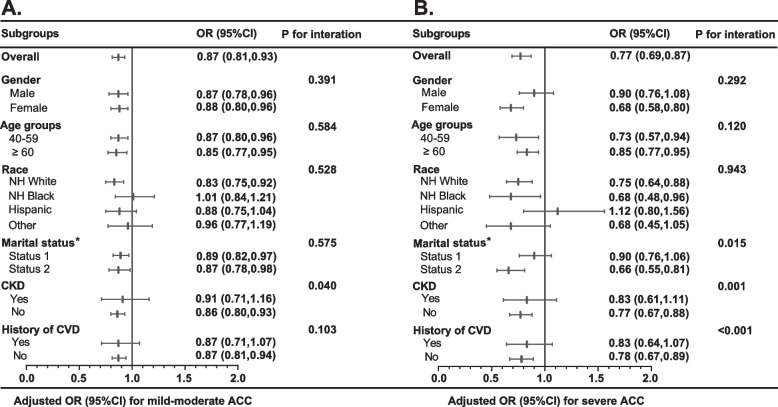
Subgroup analysis of the association of the Life’s Essential 8 score and the risk of mild-moderate (**A**) and severe (**B**) abdominal aortic calcification. Note: Logistical model adjusted for age (as a continuous variable), gender, race, poverty ratio (as a continuous variable), education levels, and marital status. * Status 1 indicates married or living with a partner, while status 2 indicates never married/widowed/divorced/separated. Abbreviation: OR, odds ratios; CI, confidence interval; NH, non-Hispanic; CKD, chronic kidney disease; CVD, cardiovascular disease

In subgroup analysis, the association between LE8 score and ACC was not consistently significant across certain groups (Fig. 3). Specifically, the relationship between LE8 score and ACC lacked statistical significance among Hispanic participants, those with CKD condition, and participants with a history of CVD (*P* > 0.05). Similar insignificance was observed for mild-moderate ACC in NH Black and Other races (*P* > 0.05). Additionally, the association between LE8 score and severe ACC was not statistically significant in participants who were married or living with a partner (*P* > 0.05). Interaction testing indicated that gender, age, race, marital status, and history of CVD did not significantly impact the association between LE8 score and mild-moderate ACC (all *P* for interaction > 0.05). However, CKD condition significantly influenced this association (*P* for interaction < 0.05). The inverse association between LE8 score and mild-moderate ACC appeared stronger in populations without CKD condition (OR for per 10 scores increase, 0.86; 95% CI 0.80, 0.93). Moreover, significant interactions between LE8 score and marital status, CKD condition, and history of CVD were observed with severe ACC (*P* < 0.05 for interaction). The inverse association between LE8 score and severe ACC appeared stronger in participants who were never married/widowed/divorced/separated (OR for per 10 scores increase, 0.66; 95% CI 0.55, 0.81), those without CKD condition (OR for per 10 scores increase, 0.77; 95% CI 0.67, 0.88), and participants without a history of CVD (OR for per 10 scores increase, 0.78; 95% CI 0.67, 0.89).

## Discussion

This study, conducted among US NHANES participants (2013–2014), affirmed our hypothesis that adults with higher levels of CVH metrics assessed by LE8 have a reduced risk of ACC. We observed a linear dose–response association between increased LE8 score and decreased ACC risk, with each 10-point rise in LE8 score associated with a 13% reduction in mild-to-moderate ACC and a 23% reduction in severe ACC.

Several previous studies have explored the connection between CVH and artery calcification. For instance, a cohort study of 65,494 adults found a correlation between higher cardiovascular health scores assessed by LF7 and a lower prevalence of coronary artery calcification [24]. Similarly, The Coronary Artery Risk Development in Young Adults (CARDIA) Study, after a 20-year follow-up, identified a link between positive CVH changes during young adulthood and a reduced risk of coronary artery calcification risk in middle age [25]. Our findings align with these studies and expand on them by utilizing new CVH metrics based on the updated LE8 assessment. The LE8 scoring system, an enhancement of the LS7 metrics proposed by the AHA in 2010, provides a more refined evaluation of cardiovascular health, considering individual behaviors and indicators with increased sensitivity [8]. Our study demonstrated that higher total CVH metrics scores were associated with a notably reduced risk of mild-to-moderate and severe ACC in a dose–response manner. Spline plots illustrated that improvements at any level of CVH scores were associated with decreased ACC risks, underscoring the significance of even marginal improvements, particularly within individuals holding CVH scores of 61.25 or higher. Additionally, the subgroup analysis revealed a more robust inverse association between the novel CVH metrics and mild-moderate ACC in participants without CKD. The negative correlation between the CVH metrics and severe ACC was more pronounced among participants without CKD, without a history of CVD, and never married/widowed/divorced/separated. Notably, the novel CVH metrics in CKD/CVD patients is susceptible to more confounding factors, such as the presence of various illnesses, accompanying poor nutritional status, and altered lifestyle [26, 27]. Consequently, the novel CVH metrics may not accurately reflect the degree of ACC in CKD/CVD patients compared to general population. Furthermore, previous studies indicate that individuals who are never married, widowed, divorced, or separated represent potentially vulnerable subgroups due to diminished social support, higher psychological burdens and an increased risk of cardiovascular diseases [28, 29]. Therefore, special attention is warranted to prevent adverse health outcomes and mitigate additional burdens on healthcare systems in the future for this specific population.

The prevalence of a high CVH score based on LE8 among US adults was found to be low, and this total CVH score is inversely correlated with all-cause and CVD-specific mortality [30, 31]. AAC, as a marker of subclinical atherosclerotic disease and an independent predictor of subsequent vascular morbidity and mortality, could be utilized to identify individuals benefiting from more aggressive cardiovascular primary prevention strategies. While our study confirms the correlation between CVH and ACC, further prospective research is necessary to determine the causal relationship between cardiovascular health evaluated based on the LE8 score and arterial calcification.

In our correlation analysis between LE8 components and AAC, nicotine exposure score, blood glucose score, and blood pressure scores emerged as primary contributors to arterial calcification. Smoking, extensively studied for its association with arterial calcification, was examined here through nicotine exposure assessed by LE8, encompassing both active smoking and secondhand smoke exposure [32–34], providing a more comprehensive and reasonable evaluation of smoking status. Elevated glucose levels have been linked to vascular smooth muscle cell calcification, contributing to diabetes-related vascular calcification [35, 36]. Our findings support a reduced risk of vascular calcification associated with optimal blood glucose levels, encompassing biochemical glucose levels, diabetic history, and medication status. Elevated blood pressure has been an independent and robust predictive factor for cardiovascular diseases, with significant correlations between systolic pressure and vascular calcification across all vessels [37]. Our detailed blood pressure assessment considered medication usage, revealing a negative correlation between blood pressure scores and calcification. Moreover, prior research has suggested obesity as a contributing factor to cardiovascular disease. However, our investigation evaluated participants’ weight status using BMI and found a positive correlation between BMI scores of LE8 and ACC risk. It’s worth noting that the association between BMI and arterial calcification has been inconsistent in previous studies [38, 39]. BMI, being an imperfect measure, does not precisely capture body fat and is unsuitable for multi-species studies [40]. Comparing our finding with previous research on this topic is rather difficult due to variations in study objectives, analytical approaches, and the diverse ethnic composition of the study populations.

Our study noted that physical activity levels and blood lipid scores were only associated with mild-to-moderate calcification and not with severe calcification. While previous studies have explored the link between physical activity and arterial calcification, our findings did not show consistent results [41, 42]. The latest research utilizing the NHANES database discovered a negative correlation between physical activity during working hours and the ACC score, while leisure-time physical activity exhibited no such association [13]. Given the inclusion of all moderate-to-vigorous physical activities, encompassing both work and leisure, consistent outcomes weren’t attained. Similarly, the relationship between blood lipid scores and severe calcification did not exhibit significant differences due to the limited number of participants with severe ACC.

Inconsistent with prior data, our study did not find an association between diet scores assessed by HEI-2015 and AAC, potentially due to scoring method variations [43]. Additionally, sleep health scores assessed by LE8 criteria did not correlate with ACC, differing from findings in previous cohort studies. Previous studies found longer sleep durations were significantly associated with decreased artery calcification, while severe obstructive sleep apnea was associated with a greater extent of AAC [12, 44]. However, the LE8 scoring system does not consider extended sleep beneficial; instead, an excessively extended sleep duration receives lower scores. These outcomes suggest that the AHA may consider reconsidering the ideal dietary or sleep health levels concerning ACC in future updates.

There are several strengths in this study. Our study was the first to explore the association of novel CVH metrics using LE8 and its components with AAC. Meanwhile, all the data we used were obtained from NHANES, which has a standardized data collection process to ensure data accuracy. Additionally, we explored the dose–response relationship between CVH and ACC, determining the minimum threshold for beneficial associations. However, we need to consider several potential limitations in our study. Firstly, although we controlled for several potential confounding factors, the cross-sectional design of our study limits establishing a causal relationship between CVH and AAC. Meanwhile, our findings distinctly indicate a correlation between better CVH and reduced ACC risk, laying the groundwork for further prospective research. Secondly, our analysis involved participants from a single nation, potentially limiting the generalizability of this study’s conclusions to many countries worldwide. Lastly, many indicators of CVH in our study relied on self-reported questionnaires, possibly introducing measurement errors and recall biases.

The findings of this study underscore the importance of public education and health campaigns emphasizing CVH concepts to promote overall health and prevent cardiovascular diseases. Enhancing public awareness of CVH concepts can elevate health consciousness and encourage proactive measures. Further research will deepen our understanding of the mechanisms and associations between cardiovascular health and arterial calcification, offering strategies for future prevention and treatment.

## Conclusions

This cross-sectional study demonstrated that the novel CVH metrics, evaluated using LE8, had an inverse association with the risk of AAC. Among the LE8 components, nicotine exposure, blood glucose, and blood pressure emerged as significant factors linked to ACC risk. These findings suggest a promising role for LE8 as a viable method to promote cardiovascular health. Additionally, these findings may support further large-scale prospective studies to clarify the precise causality of this relationship for preventing vascular calcification and CVD.

### Supplementary Information

**Supplementary Material 1.**

## Data Availability

No datasets were generated or analysed during the current study.

## Abbreviations

AAC: Abdominal aortic calcification
AHA: American Heart Association
BMI: Body mass index
BP: Blood pressure
CVH: Cardiovascular health
CVD: Cardiovascular disease
CKD: Chronic kidney disease
CI: Confidence interval
DXA: Dual-energy X-ray absorptiometry
eGFR: Estimated glomerular filtration rate
GED: General educational development test
HEI: Healthy eating index
OR: Odds ratio
PIR: Poverty-income ratio
NH: Non-Hispanic
NHANES: National Health and Nutrition Examination Survey
LE8: Life’s essential 8
LF7: Life’s simple 7
