# Supplementary material for: Associations between Life’s Essential 8 and abdominal aortic calcification among US Adults: a cross-sectional study
Source: BMC Public Health. 2024 Apr 19;24:1090. doi: 10.1186/s12889-024-18622-7 (PMC11031939; doi:10.1186/s12889-024-18622-7)
Supplement: Supplementary file 1 — Supplementary Material 1. [file 12889_2024_18622_MOESM1_ESM.docx]

**Supplemental materials**

**Associations between Life’s Essential 8 and abdominal aortic calcification among US Adults: a cross-sectional study**

**Table of Contents**

Table S12

Table S25

**Table S1. Definition and scoring approach for the American Heart Association’s Life’s Essential 8 score.**

| **Domain** | **CVH Metric** | **Measurement** | **Quantification and Scoring of CVH Metric** |
| --- | --- | --- | --- |
| **Health Behaviors** | **Diet** | Healthy Eating Index-2015 diet score percentile.  Example tools for measurement: NHANAES Dietary data | Quantiles of HEI diet adherence  Scoring (Population):  Points Quantile  100 ≥95^th^ percentile (top/ideal diet)  80 75^th^ – 94^th^ percentile  50 50^th^ – 74^th^ percentile  25 25^th^ – 49^th^ percentile  0 1^st^ – 24^th^ percentile (bottom/least ideal quartile) |
|  | **Physical activity^&^** | Self-reported minutes of moderate or vigorous physical activity per week  Example tools for measurement: NHANES PAQ-H questionnaire | Metric: Minutes of moderate (or greater) intensity activity per week  Scoring:  Points Minutes  100 ≥150  90 120 – 149  80 90 – 119  60 60 – 89  40 30 – 59  20 1 – 29  0 0 |
|  | **Nicotine exposure** | Self-reported use of cigarettes or inhaled nicotine-delivery system (NDS)  Example tools for measurement: NHANES SMQ-H questionnaire and SMQFAM-H questionnaire | Metric: combustible tobacco use and inhaled NDS use or secondhand smoke exposure  Scoring:  Points Status  100 Never smoker  75 Former smoker, quit ≥5 yrs  50 Former smoker, quit 1 - <5 yrs  25 Former smoker, quit <1 year, or currently using inhaled NDS  0 Current smoker  Subtract 20 points (unless the score is 0) for living with an active indoor smoker in the home. |
|  | **Sleep health** | Self-reported average hours of sleep per night  Example tools for measurement: NHANES SLQ-H questionnaire | Metric: Average hours of sleep per night  Scoring:  Points Level  100 7 – <9  90 9 – <10  70 6 – <7  40 5 – <6 or ≥10  20 4 – <5  0 <4 |
| **Health Factors** | **Body mass index** | Body weight (kg) divided by height squared (m^2^)  Example tools for measurement: NHANES SLQ-H questionnaire | Metric: Body mass index (kg/m^2^)  Scoring:  Points Level  100 <25  70 25.0 – 29.9  30 30.0 – 34.9  15 35.0 – 39.9  0 ≥40.0 |
|  | **Blood lipids** | Plasma total and HDL cholesterol with calculation of non-HDL cholesterol  Example tools for measurement: NHANES HDL-H / TCHOL-H data and RXQ_RX_H questionnaire | Metric: Non-HDL cholesterol (mg/dL)  Scoring:  Points Level  100 <130  60 130 – 159  40 160 – 189  20 190 – 219  0 ≥220  If the drug-treated level, subtract 20 points. |
|  | **Blood glucose^*^** | Fasting blood glucose (FBG) or casual hemoglobin A1c (HbA1c)  Example tools for measurement: NHANES GHB_H/ GLU_H data and DIQ_H/ RXQ_RX_H questionnaire | Metric: Fasting blood glucose (mg/dL) or HbA1c (%)  Scoring:  Points Level  100 No history of diabetes and FBG <100 (or HbA1c < 5.7)  60 No diabetes and FBG 100-125 (or HbA1c 5.7-6.4) (prediabetes)  40 Diabetes with HbA1c <7.0  30 Diabetes with HbA1c 7.0 – 7.9  20 Diabetes with HbA1c 8.0 – 8.9  10 Diabetes with Hb A1c 9.0 – 9.9  0 Diabetes with HbA1c ≥10.0 |
|  | **Blood pressure^#^** | Appropriately measured systolic and diastolic blood pressure  Example tools for measurement: NHANES BPX_H data and RXQ_RX_H questionnaire | Metric: Systolic and diastolic blood pressure (mm Hg)  Scoring:  Points Level  100 <120/<80 (optimal)  75 120-129/<80 (elevated)  50 130-139 or 80-89  25 140-159 or 90-99  0 ≥160 or ≥100  Subtract 20 points if treated level. |

^&^Thresholds are based in part on US Physical Activity Guidelines. Each minute of moderate activity should count as 1 minute, and each minute of vigorous activity should count as 2 minutes toward the total for the week.

**^*^**If an individual patient with prediabetes (i.e., not yet diagnosed formally with diabetes) is being treated with metformin to prevent the onset of diabetes and has normoglycemic levels, subtract 20 points.

^#^Blood pressure was calculated from each respondent's available measurements

using the NHANES recommended method (https://wwwn.cdc.gov/Nchs/Nhanes/1999-2000/BPX.htm).

**Table S2. Association of the Life’s Essential 8 components with** **abdominal aortic calcification (AAC).**

| **Variables** | **Mild–moderate AAC versus no AAC** | | **Severe AAC**  **versus no AAC** | |
| --- | --- | --- | --- | --- |
|  | **OR (95% CI)** | **P value** | **OR (95% CI)** | **P value** |
| **Unadjusted** | | | | |
| Diet score, per 10 points increase | 0.99 (0.96,1.02) | 0.474 | 0.99 (0.95,1.03) | 0.620 |
| Physical activity score, per 10 points increase | 0.96 (0.94,0.98) | <0.001 | 0.97 (0.94,0.99) | 0.021 |
| Nicotine exposure score, per 10 points increase | 0.96 (0.94,0.99) | 0.005 | 0.97 (0.93,1.00) | 0.056 |
| Sleep health score, per 10 points increase | 1.01 (0.97,1.05) | 0.745 | 1.09 (1.02,1.15) | 0.008 |
| Body mass index score, per 10 points increase | 1.05 (1.02,1.09) | 0.001 | 1.09 (1.04,1.14) | <0.001 |
| Blood lipid score, per 10 points increase | 0.97 (0.94,1.00) | 0.065 | 1.00 (0.96,1.05) | 0.89 |
| Blood glucose score, per 10 points increase | 0.95 (0.91,0.98) | 0.002 | 0.86 (0.82,0.90) | <0.001 |
| Blood pressure score, per 10 points increase | 0.93 (0.90,0.96) | <0.001 | 0.82 (0.79,0.86) | <0.001 |
| **Model^*^** | | | | |
| Diet score, per 10 points increase | 0.99 (0.95,1.02) | 0.507 | 0.95 (0.90,1.01) | 0.110 |
| Physical activity score, per 10 points increase | 0.97 (0.95,0.99) | 0.007 | 1.01 (0.97,1.05) | 0.710 |
| Nicotine exposure score, per 10 points increase | 0.95 (0.93,0.98) | <0.001 | 0.87 (0.83,0.92) | <0.001 |
| Sleep health score, per 10 points increase | 0.99 (0.95,1.04) | 0.764 | 1.02 (0.95,1.10) | 0.535 |
| Body mass index score, per 10 points increase | 1.06 (1.02,1.09) | 0.001 | 1.11 (1.05,1.18) | <0.001 |
| Blood lipid score, per 10 points increase | 0.96 (0.92,0.99) | 0.013 | 0.99 (0.93,1.05) | 0.687 |
| Blood glucose score, per 10 points increase | 0.96 (0.93,1.00) | 0.071 | 0.87 (0.81,0.92) | <0.001 |
| Blood pressure score, per 10 points increase | 0.97 (0.94,1.00) | 0.066 | 0.90 (0.86,0.95) | <0.001 |

Abbreviation: OR: odds ratio, CI: confidence interval, AAC: abdominal aortic calcification.

^*^Adjusted for age (as a continuous variable), sex, race/ethnicity, poverty ratio (as a continuous variable), education levels, marital status and other components of the Life’s Essential 8.
